# Association of Burden and Prevalence of Arthritis With Disparities in Social Risk Factors, Findings From 17 US States

**DOI:** 10.5888/pcd19.210277

**Published:** 2022-02-17

**Authors:** Zachary D. Rethorn, Timothy J. Rethorn, Chad E. Cook, Jason A. Sharpe, S. Nicole Hastings, Kelli D. Allen

**Affiliations:** 1 Doctor of Physical Therapy Division, Duke University School of Medicine, Durham, North Carolina; 2 Center of Innovation to Accelerate Discovery and Practice Transformation, Veterans Affairs Health Care System, Durham, North Carolina; 3 School of Health and Rehabilitation Sciences, The Ohio State University, Columbus, Ohio; 4 Duke Clinical Research Institute, Duke University School of Medicine, Durham, North Carolina; 5 Department of Population Health Sciences, Duke University Medical Center, Durham, North Carolina; 6 Department of Medicine, Duke University Medical Center, Durham, North Carolina; 7 Geriatric Research, Education, and Clinical Center, Veterans Affairs Health Care System, Durham, North Carolina; 8 Center for the Study of Human Aging and Development, Duke University School of Medicine, Durham, North Carolina; 9 Department of Medicine and Thurston Arthritis Research Center, University of North Carolina, Chapel Hill, North Carolina

## Abstract

**Introduction:**

Social risks previously have been associated with arthritis prevalence and costs. Although social risks often cluster among individuals, no studies have examined associations between multiple social risks within the same individual. Our objective was to determine the association between individual and multiple social risks and the prevalence and burden of arthritis by using a representative sample of adults in 17 US states.

**Methods:**

Data are from the 2017 Behavioral Risk Factor Surveillance System. Respondents were 136,432 adults. Social risk factors were food insecurity, housing insecurity, financial insecurity, unsafe neighborhoods, and health care access hardship. Weighted χ^2^ and logistic regression analyses, controlling for demographic characteristics, measures of socioeconomic position, and other health conditions examined differences in arthritis prevalence and burden by social risk factor and by a social risk index created by summing the social risk factors.

**Results:**

We observed a gradient in the prevalence and burden of arthritis. Compared with those reporting 0 social risk factors, respondents reporting 4 or more social risk factors were more likely to have arthritis (adjusted odds ratio [AOR], 1.92; 95% CI, 1.57–2.36) and report limited usual activities (AOR, 2.97; 95% CI, 2.20–4.02), limited work (AOR, 2.72; 95% CI, 2.06–3.60), limited social activities (AOR, 3.10; 95% CI, 2.26–4.26), and severe joint pain (AOR, 1.86; 95% CI, 1.44–2.41).

**Conclusion:**

Incremental increases in the number of social risk factors were independently associated with higher odds of arthritis and its burden. Intervention efforts should address the social context of US adults to improve health outcomes.

**SUMMARY**

**What is already known on this topic?**

Social risks have been previously associated with arthritis prevalence and burden. Although social risks often cluster among individuals, no studies have examined associations between multiple social risks within the same individual.

**What is added by this report?**

Incremental increases in the number of social risk factors were independently associated with higher odds of arthritis and its burden.

**What are the implications for public health practice?**

Targeting individuals with multiple social risk factors may help reduce the prevalence and burden of arthritis among vulnerable populations.

## Introduction

Approximately 1 in 4 US adults have medically diagnosed arthritis, which is the most common cause of disability in the US, with over $300 billion in costs in 2013 (1,2). Although no cures exist for arthritic conditions, a number of biomedical and behavioral factors can modify the prevalence and burden of arthritis; these include obesity and physical activity (3). However, these traditionally measured factors fail to adequately explain patients’ risk of developing arthritis and the burden of arthritis (ie, mortality, morbidity, or financial cost).

In recent years, public policy groups who engage in community health care and cost management have increasingly recognized the influence of health risks related to social context (4). Whereas clinical care pathways for arthritis commonly consider biological and psychological factors (5), social determinants of health (SDOH) are not routinely taken into account. SDOH are broadly defined as the conditions in which people are born, work, live, and play and include areas such as economic stability, education, social and community context, and the built environment (4). Social risk factors are individual-level adverse SDOH that can be identified through screening tools (eg, positive for housing insecurity), although social needs are social risks prioritized by patients (eg, request for housing assistance) (6). Because arthritis is so prevalent and costly, it is essential that the influence of social risk factors on arthritis is better understood.

Studies have quantified the relative contribution of traditional biomedical factors and social influences such as education and income to the development and burden of arthritis (7). However, social risk factor variables do not routinely exist alone and may cluster cross-sectionally. Shifting from a single risk factor analysis to a more comprehensive perspective that seeks to understand the complexity of coexisting social risk factors may offer greater insight into the effect of the accumulation of influences on health (8). No studies to date, however, have examined the effect of multiple or coexisting social risk factors on arthritis prevalence and burden. Our aim was to explore the association between social risk factors and the prevalence and burden of arthritis individually and with a social risk index based on the total number of social risk factors reported. A social risk index is a composite statistic that measures changes in a representative group of systemic social issues, or a compounding measure that aggregates multiple indicators (9).

## Methods

This study adheres to REporting of studies Conducted using Observational Routinely collected Data (RECORD) guidelines (10). No patients were involved in the design or conduct of this study. The study was approved by the Institutional Review Board at Duke University.

### Study design and participants

Our study was a cross-sectional analysis of the Centers for Disease Control and Prevention (CDC) 2017 Behavioral Risk Factor Surveillance System (BRFSS) survey from 17 US states that administered the SDOH module to a total of 136,432 respondents. We analyzed the data in October 2020. The BRFSS is an annual, nationally representative computer-assisted telephone survey of health-related risk behaviors, health conditions, and use of preventive services among noninstitutionalized adults, aged 18 years or older. The median response rate in 2017 in this sample for telephone and cellular telephone respondents combined was 46.5% (range, 32.8%–64.1%) (11). Of the states that administered the SDOH module in 2017, 13 (Florida, Georgia, Iowa, Kentucky, Massachusetts, Minnesota, Mississippi, New Hampshire, Pennsylvania, Utah, West Virginia, Wisconsin, and Wyoming) administered the module to their entire sample, whereas 4 (Colorado, Maryland, Ohio, and Oklahoma) administered the module to 1 or more splits in their samples. Split samples maximize information gathering while reducing survey fatigue by dividing the sample into equivalent probability-based samples. We merged the sample split data sets with the national BRFSS data sets by using publicly available 2017 BRFSS data files from CDC. More information about split sample methods and use is available from CDC (12).

### Social risk factor predictor variables

Based on the Commission on Social Determinants of Health Final Report published by the World Health Organization (4), we selected all 6 questions from the BRFSS SDOH module and 1 question on health care access from the core BRFSS survey. We combined these variables to create 5 social risk factors: food insecurity, housing insecurity, financial insecurity, unsafe neighborhood, and health care access hardship. These variables were selected because addressing the social risks they represent creates substantial opportunities to address the social determinants of arthritis outcomes (4). A positive response for food insecurity was identified when a participant responded “often true” or “sometimes true” to the statements, “The food that I bought just didn’t last, and I didn’t have money to get more” and “I couldn’t afford to eat balanced meals.” A positive response for housing insecurity was identified when a participant responded yes to the question, “During the last 12 months, was there a time when you were not able to pay your mortgage, rent, or utility bills?” A positive response for financial insecurity was identified when a participant responded, “[We did] not have enough money to make ends meet” or “[We] have just enough money to make ends meet” to the question, “In general, how do your finances usually work out at the end of the month?” A positive response to unsafe neighborhood was identified when a participant reported “unsafe” or “extremely unsafe” to the question, “How safe from crime do you consider your neighborhood to be?” A positive response for health care access hardship was identified when a participant responded yes to the question, “Was there a time in the past 12 months when you needed to see a doctor but could not because of cost?” Each response was dichotomized to indicate exposure. Positive responses to each social risk factor were summed to create a social risk index. Social risk index scores ranged from 0 (no social risk factors reported) to 4 or more (a positive response to each social risk factor reported). Higher scores indicated increasing numbers of social risk factors.

### Arthritis-related outcome variables

To identify the prevalence of arthritis, we used an umbrella designation reflective of diagnostic conditions. For the BRFSS, arthritis is defined a yes response to the question, “Have you ever been told by a doctor or other health care professional that you have arthritis, rheumatoid arthritis, gout, lupus, or fibromyalgia?” This question has been in use since 2002 and was designed to incorporate elements of the 1994 public health definition of arthritis developed by the National Arthritis Data Workgroup (13). All 4 questions from the arthritis burden module were used to identify burden. Limited activities were defined as a yes response to the question, “Are you now limited in any way in any of your usual activities because of arthritis or joint symptoms?” Limited work was defined as a response of yes to the question, “Do arthritis or joint symptoms now affect whether you work, the type of work you do, or the amount of work you do?” Limited social activities was defined as the response of yes to the question, “During the past 30 days, to what extent has your arthritis or joint symptoms interfered with your normal social activities, such as going shopping, to the movies, or to religious or social gatherings?” Respondents with severe joint pain were described by those whose responses ranged from 7 to 10 to the question, “Please think about the past 30 days, keeping in mind all of your joint pain or aching and whether or not you have taken medication. On a scale of 0 to 10 where 0 is no pain or aching and 10 is pain or aching as bad as it can be, during the past 30 days, how bad was your joint pain on average?” This description for severe joint pain has been used in previous public health surveillance, and the BRFSS has been extensively validated (14,15).

### Covariates

We selected covariates on the basis of their potential to influence the predictor or outcome (16). Demographic characteristics were age, sex, race, ethnicity, marital status, and health insurance. Measures of socioeconomic position were educational attainment, income, current employment status, and home ownership and were included because they are conceptually linked with social risk factors. Health conditions were heart attack, coronary heart disease, stroke, asthma, skin cancer, other cancer, chronic obstructive pulmonary disease, depression, chronic kidney disease, obesity, and multimorbidity (defined as the presence of at least 2 of the aforementioned health conditions). We included these health conditions because arthritis often co-occurs with other conditions (17).

### Statistical analysis

Descriptive statistics for the overall sample and the social risk index were analyzed for demographic characteristics, measures of socioeconomic position, and health conditions. Colinearity among predictor social risk variables was assessed using the φ (phi) coefficient. We defined a correlation of 0.50 or more as indicative of colinearity. Prevalence and 95% CIs of each of the 5 selected social risk factors and the social risk index were calculated. Weighted Wald χ^2^ analyses were used to calculate bivariate associations between arthritis prevalence and burden and between each social risk factor and social risk index score. Multivariable weighted logistic regression models adjusted for all covariates were used to examine the association of arthritis prevalence and burden with each social risk factor and the social risk index score. Multicolinearity was assessed for each model by using variance inflation factors. We defined a variance inflation factor of 3.0 or more as indicative of multicolinearity. Significance was set at *P* < .05. Analyses were performed using R version 4.0.2 (R Foundation for Statistical Computing) and weighted to account for the complex sampling design and nonresponses. We report weighted percentages and weighted adjusted odds ratios with 95% CIs.

## Results

### Sample

Weighted estimates of demographics, measures of socioeconomic position, and health conditions for the full sample (N = 136,432) and each of the social risk factors are presented. (Table 1). All ages were well represented, and respondents were predominantly White (70.9%), had an annual income of $50,000 or more (49.0%), and had at least a high school diploma (88.1%). The percentage of respondents residing in the West was 8.4%, whereas 47.4% resided in the South.

**Table 1:** Demographic Characteristics of Individuals from 17 States in the 2017 BRFSS Sample^a^

Characteristic	0 of 5 social risk factors^b^ (54.3%)	1 of 5 social risk factors^b^ (25.0%)	2 of 5 social risk factors^b^ (11.2%)	3 of 5 social risk factors^b^ (6.3%)	≥4 of 5 social risk factors^b^ (3.2%)	Sample totals (100%)
**Age group, y**						
18–24	11.5	12.3	15.8	12.8	12.2	12.3
25–34	15.3	16.2	19.4	22.0	21.6	16.6
35–44	15.2	14.8	16.4	20.7	22.8	15.8
45–54	16.0	16.8	17.3	17.1	20.4	16.5
55–64	17.1	17.0	16.6	17.9	16.9	17.0
≥65	24.9	22.9	14.5	9.5	6.1	21.7
**Sex**						
Male	51.0	47.3	45.5	41.4	41.3	48.5
Female	49.0	52.7	54.5	58.6	58.7	51.5
**Married or partnered**	61.7	52.7	43.4	41.0	36.1	55.3
**Race or ethnicity**						
African American	10.0	13.0	18.7	20.3	22.7	12.8
Hispanic	7.6	12.2	16.0	15.8	15.4	10.5
Multiracial	1.0	1.3	1.7	2.1	2.6	1.3
Other	4.6	4.6	4.4	4.1	4.0	4.5
White	76.8	68.8	59.2	57.7	55.3	70.9
**Household income, $**						
<15,000	4.4	9.3	18.8	24.1	29.0	9.4
15,000–24,999	10.1	20.5	27.9	35.2	34.4	17.1
25,000-34,999	8.0	13.5	15.2	13.7	14.2	10.8
35,000-49,999	13.0	15.4	14.7	12.4	11.2	13.7
≥50,000	64.5	41.3	23.4	14.6	11.2	49.0
**Homeowners**	77.4	67.9	53.9	46.0	40.3	69.1
**Educational attainment**						
Less than high school graduation	7.8	14.1	18.9	20.9	21.8	11.9
High school diploma or GED	27.8	31.9	34.6	33.4	33.3	30.1
Some college	30.1	30.6	31.8	33.8	33.7	30.8
College degree	34.3	23.3	14.7	11.9	11.2	27.2
**Employment status**						
Employed	60.3	55.3	51.5	49.6	51.3	57.1
Unemployed	3.5	5.9	8.9	12.7	14.6	5.6
Unable to work	3.4	31.5	13.9	18.5	14.1	7.1
Other	32.8	7.3	25.7	19.2	20.0	30.2
**Census region**						
Midwest	24.6	24.0	23.6	23.4	22.5	24.2
Northeast	21.2	19.5	18.4	17.4	13.7	20.0
South	45.9	48.1	48.8	51.1	55.7	47.4
West	8.3	8.4	9.2	8.1	8.1	8.4
**Has health insurance**	93.6	88.0	81.4	76.4	69.7	90.0
**Health conditions**						
Heart attack	3.8	5.0	5.7	6.3	7.4	4.6
Coronary heart disease	3.9	4.9	4.7	5.3	6.8	4.4
Stroke	2.6	3.7	4.9	5.6	6.8	3.4
Asthma	11.5	14.3	18.7	21.4	24.9	14.0
Skin cancer	7.8	6.5	4.5	3.9	2.9	6.7
Other cancer	7.2	7.4	6.3	6.9	9.3	7.2
COPD	5.0	7.4	10.7	13.0	16.7	7.1
Depression	13.5	20.0	26.8	41.1	47.2	19.4
Chronic kidney disease	2.3	3.1	3.9	5.2	5.5	3.0
Obesity	64.1	67.0	67.9	68.6	69.8	65.7
Multimorbidity (≥2 comorbidities)	30.9	38.0	44.0	52.5	58.7	36.4

Abbreviations: BRFSS, Behavioral Risk Factor Surveillance System; COPD, chronic obstructive pulmonary disease.

a Data are presented as weighted percentages of study participants.

b Social risk factors are 1) having arthritis, 2) limited activities, 3) limited work, 4) limited social activities, and 5) severe joint pain.

Overall, 54.3% (95% CI, 53.8%–54.8%) of the respondents reported experiencing none of the 5 social risk factors, 25.0% (95% CI, 24.5%–25.4%) reported 1 social risk factor, 11.2% (95% CI, 10.9%–11.6%) reported 2, 6.3% (95% CI, 6.0%–6.6%) reported 3, 2.8% (95% CI, 2.6%–2.9%) reported 4, and 0.4% (95% CI, 0.3%-0.5%) reported all 5. Mean social risk index scores were higher among women and young to middle-aged adults. Respondents identifying as multiracial, Black, and Hispanic had the highest mean social risk index scores, compared with "other" and White groups. 

### Bivariate associations of social risk factors and arthritis prevalence and burden

No evidence of colinearity was found among social risk factors. The prevalence of reporting arthritis, being limited in activities, limited work, limited social activities, or severe joint pain was significantly higher among those reporting each social risk factor individually (Table 2). In models adjusted for demographic characteristics, socioeconomic position, and health conditions, the presence of each social risk factor was associated with each outcome. Adjusted odds ratios ranged from 1.20 (95% CI, 1.11–1.29) to 2.33 (95% CI, 1.98–2.74) for the 5 social risk factors (Table 3). No evidence of multicolinearity was found.

**Table 2:** Relative Arthritis Prevalence and Burden by Individual Social Risk Factor and Social Risk Index^a^

Social risk	Total sample	Having arthritis	Limited activities	Limited work	Limited social activities	Severe joint pain
**Social risk factor**						
Food insecurity	22.2	24.3^b^	32.9^b^	38.8^b^	35.6^b^	42.0^b^
Housing insecurity	9.6	12.8^b^	18.2^b^	23.5^b^	20.0^b^	23.9^b^
Financial insecurity	47.4	53.6^b^	63.1^b^	69.5^b^	67.0^b^	72.9^b^
Unsafe neighborhood	5.6	6.8^b^	8.8^b^	10.2^b^	9.5^b^	11.2^b^
Health care access hardship	13.0	14.4^b^	20.0^b^	24.5^b^	21.2^b^	23.8^b^
** Social risk index, No. (total number of social risk factors)**						
0	54.2	49.0^b^	40.0^b^	33.9^b^	36.8^b^	31.9^b^
1	25.0	25.9	26.1	26.2	26.9^b^	26.6
2	11.2	12.5^b^	15.4^b^	17.3^b^	16.3^b^	18.4^b^
3	6.3	8.1^b^	11.5^b^	13.3^b^	12.4^b^	14.2^b^
≥4	3.3	4.5^b^	7.0^b^	9.3^b^	7.6^b^	8.9^b^

a Data are presented as weighted percentages of study participants.

b *P* < .05.

**Table 3:** Adjusted Odds Ratios^a^ for Arthritis Prevalence and Associated Burden by Social Risk

Social risk	Having arthritis	Limited activities	Limited work	Limited social activities	Severe joint pain
**Social risk factor **					
Food insecurity	1.32 (1.20–1.45)	1.65 (1.43–1.91)	1.56 (1.36–1.79)	1.73 (1.50–2.00)	1.67 (1.44–1.93)
Housing insecurity	1.59 (1.41–1.80)	1.54 (1.28–1.86)	1.76 (1.48–2.09)	1.74 (1.44–2.12)	1.59 (1.33–1.89)
Financial insecurity	1.20 (1.11–1.29)	1.53 (1.37–1.71)	1.48 (1.31–1.66)	1.64 (1.47–1.83)	1.44 (1.26–1.64)
Unsafe neighborhood	1.38 (1.15–1.64)	1.37 (1.06–1.79)	1.42 (1.11–1.82)	1.37 (1.06–1.78)	1.34 (1.06–1.70)
Health care access hardship	1.48 (1.34–1.63)	2.00 (1.72–2.33)	1.97 (1.70–2.28)	2.33 (1.98–2.74)	1.71 (1.46–1.99)
**Social risk index, No. (total number of social risk factors) **					
0	1.00 [Reference]	1.00 [Reference]	1.00 [Reference]	1.00 [Reference]	1.00 [Reference]
1	1.12 (1.05–1.20)	1.31 (1.18–1.46)	1.31 (1.17–1.47)	1.44 (1.29–1.60)	1.25 (1.10–1.43)
2	1.35 (1.21–1.50)	1.72 (1.47–2.02)	1.74 (1.49–2.04)	1.99 (1.69–2.33)	1.72 (1.46–2.02)
3	1.72 (1.50–1.98)	2.31 (1.85–2.89)	1.81 (1.49–2.20)	2.40 (1.89–3.04)	1.78 (1.45–2.18)
≥4	1.92 (1.57–2.36)	2.97 (2.20–4.02)	2.72 (2.06–3.60)	3.10 (2.26–4.26)	1.86 (1.44–2.41)

a Data are presented as weighted odds ratios (95% CI), adjusted for demographic characteristics (age, sex, race, ethnicity, marital status, and health insurance), socioeconomic position (educational attainment, income, current employment status, and home ownership), and health conditions (heart attack, coronary heart disease, stroke, asthma, skin cancer, other cancer, chronic obstructive pulmonary disease (COPD), depression, chronic kidney disease, obesity, and multimorbidity).

### Social risk index

**Figure 1:**
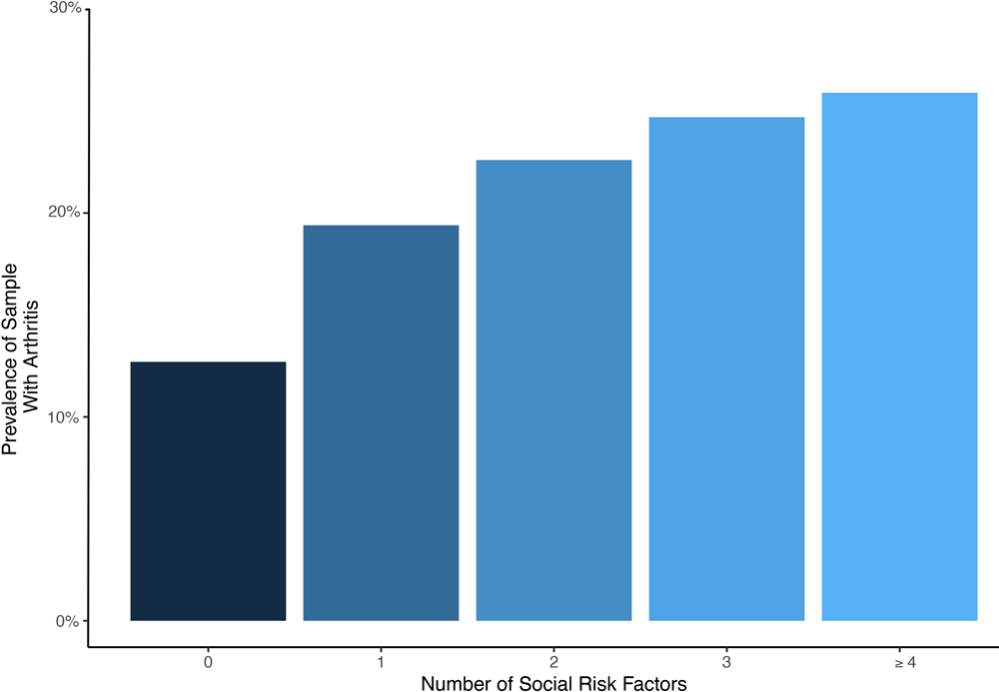
Weighted absolute prevalence of arthritis by number of social risk factors in the 2017 BRFSS sample. The prevalence of arthritis increases linearly as the number of social risk factors increase.

**Figure 2:**
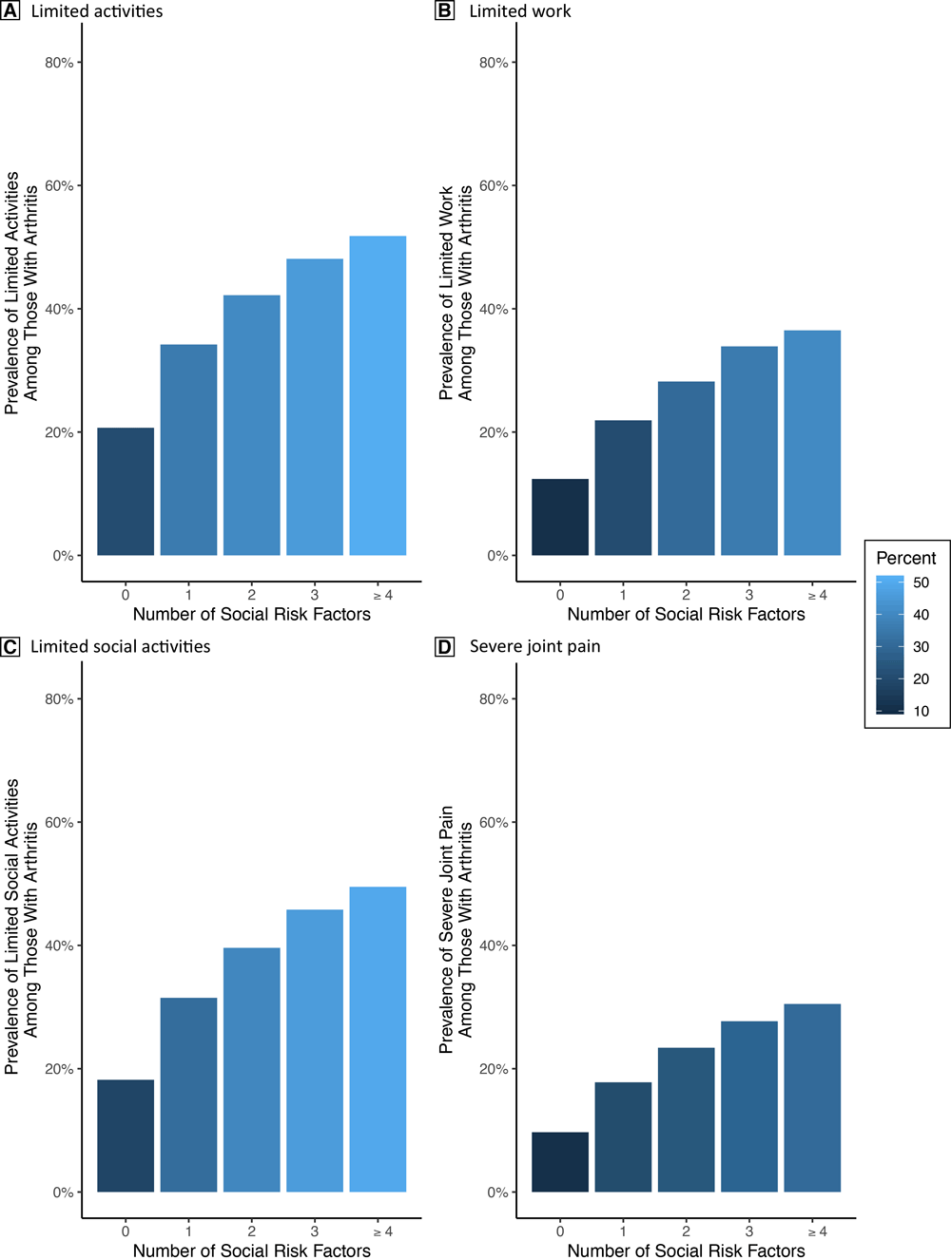
the weighted absolute prevalence of each health outcome among BRFSS participants with arthritis, 2017 BRFSS sample. The prevalence of each outcome increases linearly as the number of social risk factors increase.

The prevalence of each outcome increased with the number of social risk factors reported. Presented are the absolute prevalence of arthritis (Figure 1) and burden by number of social risk factors (Figure 2). 

**Table:** 

Number of Social Risks	Arthritis %
0	12.7
1	19.4
2	22.6
3	24.7
≥4	25.9

**Table:** 

Limited Activities, %	Limited Work, %	Limited Social Activities, %	Severe Joint Pain, %
20.7	12.4	18.2	9.7
34.2	21.9	31.5	17.8
42.2	28.2	39.6	23.4
48.1	33.9	45.8	27.7
51.8	36.5	49.5	30.5

In models adjusted for demographic characteristics, socioeconomic position, and health conditions, the gradient between each outcome and the number of associated social risk factors increased. Adjusted odds ratios ranged from 1.86 (95% CI, 1.44−2.41) to 3.10 (95% CI, 2.26−4.26) when respondents reported 4 or more social risk factors (Table 3). No evidence of multicolinearity was reported.

## Discussion

We observed a report of prevalence of arthritis and burden among individuals with social risk factors. Specifically, we noted a gradient in the social risk index where, for each additional social risk factor reported, the odds of a respondent reporting having arthritis, being limited in activities, work, and social activities, and reporting severe joint pain also increased. Associations persisted after controlling for measures of social context including demographic characteristics, measures of socioeconomic position, and the presence of other health conditions.

Our study contributes to the literature on arthritis and social risks in several ways. This study is, to our knowledge, the largest to examine the prevalence of arthritis and its associated burden by social risk factors and the first to examine the impact of co-occurring social risks on arthritis. We defined our social risk factors as a combination of individual and neighborhood-level factors and evaluated their combined influence on arthritis prevalence and burden. In contrast, many previous studies limited their perspective on social risks to only socioeconomic factors (18–20).

Considering the increasing prevalence and the substantial burden of arthritis among US adults, understanding the relationships between social risks and arthritis is an important public health effort (1). Our findings suggest that the social risk factors measured have a relationship with arthritis independent of socioeconomic position and known risk factors such as age, sex, employment, body mass index, and race or ethnicity (21,22). These factors represent modifiable social contexts that can be influenced directly through public policy, improved access to health care, and clinical programs (23,24). Furthermore, mechanisms underlying these relationships might allow mediation through other health behaviors such as physical activity, diet, and sleep (25,26). Additionally, social support and psychological state are likely proximal to health outcomes and present opportunities to develop interventions to reduce the burden of arthritis. 

Identifying the association between social risk factors and arthritis aids understanding the social contribution to the prevalence and burden of arthritis. These findings may provide additional momentum for health systems and organizations not yet addressing social risks. Because as much as 80% of the factors that influence health are outside the traditional health care system, unmet social needs may limit an organization’s ability to achieve quality benchmarks associated with value-based payment. Though some health systems are making investments to address social risk factors (27), the aggregate benefit of these investments may not exceed the cost to primary participants in community health, thus limiting the funding needed to address social risks (28). Spreading the cost and the financial benefits of interventions to address social risk shows potential ways to increase uptake among health care systems and managed care organizations (28).

Because data on key markers of social risk are sparse in many organizations, proxies such as the percentage of a clinician’s patient population that is dually enrolled in Medicare and Medicaid are commonly used. However, because Medicaid eligibility varies from state to state and dual-enrollees’ health and risk profiles vary from state to state (29), we see potential value in operationalizing social risks as a count for risk-adjustment purposes. In clinical settings, a count of social risks can be calculated and interpreted easily by using social risk screening tools.

Implementing policies to minimize the exposure of disadvantaged populations to social risk factors, and thus reducing potential vulnerability, is urgently needed (4). Including screening for social risk factors in care pathways may be one way to reduce the unequal prevalence and burden of arthritis (30). Previous studies have identified increased referral to and use of wraparound services including clinicians, such as social workers, dieticians, and behavioral health clinicians when such pathways are implemented (24). Including social risk factors in stepped care models may also be an important action toward improving the equity of arthritis-related health outcomes (5).

Our study is limited by its use of cross-sectional data from the BRFSS; therefore, causality cannot be established. It is possible that the presence of functional limitations could exacerbate social vulnerabilities. For example, pain related to arthritis may be related to the ability to work, and financial insecurity might be a result of a decreased ability to work and increasing care needs. Our inability to include this variable in analyses may have altered our results. Because as much as 80% of the factors that influence health are outside the traditional health care system, unmet social needs may limit an organization’s ability to achieve quality benchmarks associated with value-based payment. Other social risks known to be associated with arthritis, such as perceived discrimination, were not available in the BRFSS. BRFSS relies on self-reported information and retrospective reporting of social risks that can introduce memory and response biases. Our findings may not be representative of all adults in the US, as only 17 states chose to include the SDOH module in 2017, and 10 of those states had a proportion of persons living in poverty below the national average that year (31).

Social risk factors individually and concurrently according to a social risk index are associated with disparities in arthritis prevalence and burden, as those with 4 or more social risk factors had nearly twice the odds of having arthritis as those with no risk factors. Future research focusing on determining mechanisms that underlie these relationships is warranted. Results from our study inform the need for those engaged in clinical care and policy making to consider the social environment of individuals with arthritis. Reducing the prevalence and burden of arthritis by addressing social determinants is warranted.
